# Supplementary material for: African Genomic Medicine Portal: A Web Portal for Biomedical Applications
Source: J Pers Med. 2022 Feb 11;12(2):265. doi: 10.3390/jpm12020265 (PMC8879570; doi:10.3390/jpm12020265)
Supplement: Supplementary file 1 [file jpm-12-00265-s001.zip › jpm-1534466-supplementary.pdf]

Supplementary Materials for: African Genomic Medicine Portal: A Web Portal for Biomedical Applications

Supplementary Materials File S1

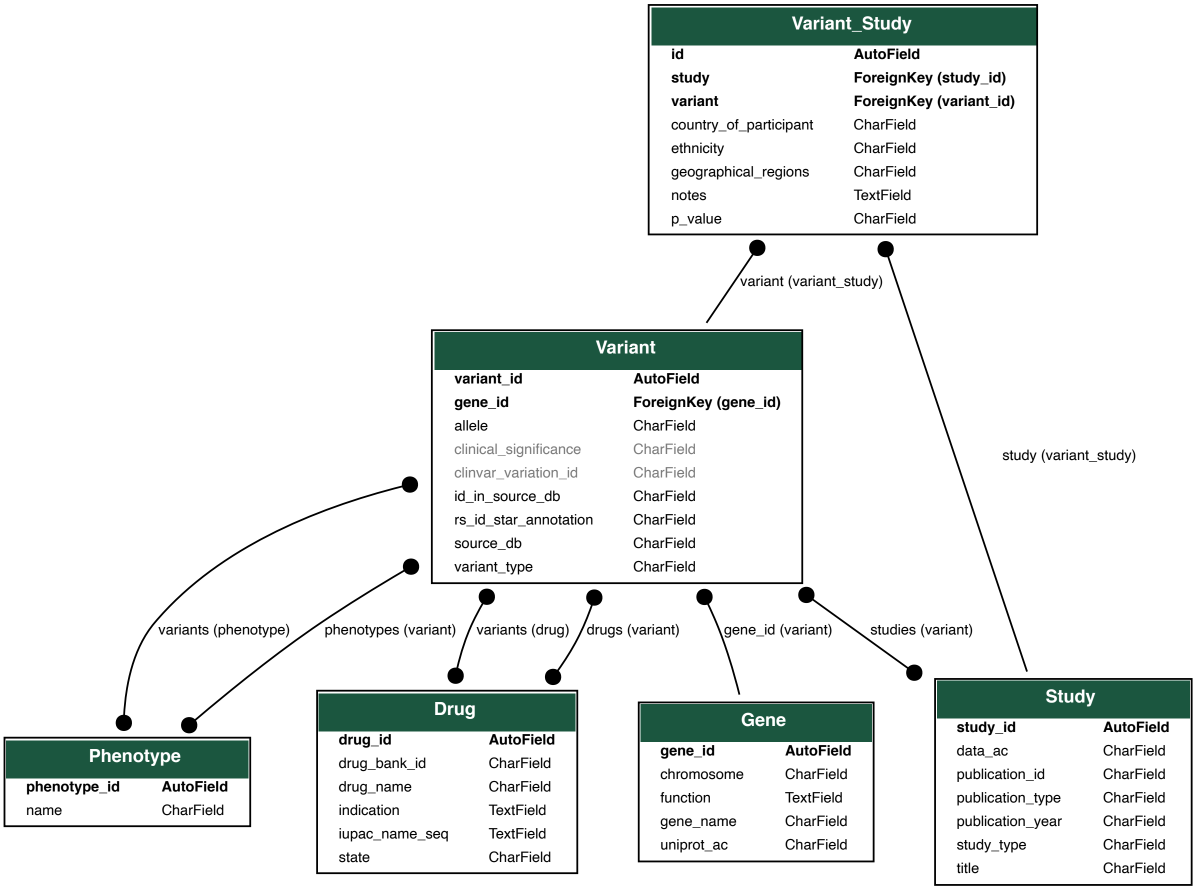

Figure S1: Entity Relationship Model for the AGMP.

Supplementary Materials File S2

Description of the attributes in the AGMP database.

Table S1: Description of the attributes in the AGMP database.

**Table**	**Attribute**	**Description**
Variant_Study	Variant	Refers to the RS identifier in dbSNP or the star allele nomenclature
	Country of participant	Is the country or countries from which the participants in a study were recruited. In the case where the country is not available, annotating with a broader geographical region can be made (e.g. Sub-Saharan Africa).
	Ethnicity	Ethnicity reported by the authors of the study
	Geographical region	Refers to the geographical origin of the individuals in the study only with African ancestry. The regions cover North, East, West, South Africa and the African-American and Afro-Caribbean individuals. For studies that do not provide the country from which the cohort was collected, general terms are used such as Sub-Saharan Africa.
	P-value	The p-value is reported for the population specified in the Geographical region field. If multiple p-values are calculated for different populations in the same study, only those for populations of African Ancestry are reported.
	Notes	Includes other information mentioned in the associated reference such as the size of the cohort or the disambiguation.
Variant	Gene ID	Corresponds to the gene symbol
	Allele	The allele responsible for the observed phenotype. 9
	Clinical significance	Reports the clinical impact annotated in the ClinVar database of the allele causing or associated with the phenotype
	Clinvar variation id	Is the ClinVar accession for annotation of the allele causing or associated with the phenotype
	ID in source database	Is the accession ID in PharmGKB or DisGeNET
	Source database	PharmGKB or DisGeNET
	Variant type	Can be either a SNV or a star allele variant
Phenotype	Phenotype name	Refers to the effect on the drug-response or the disease
Gene	Chromosome	Chromosome location of the variant
	Function	Describes the biological function for the corresponding gene retrieved from the UniProt database
	UniProt	Corresponds to the UniProt accession of the protein encoded by the annotated gene
	gene_name	Gene symbol according to the Human Genome Organisation
Drug	DRUGBANK ID	Is the identifier of the drug in the DRUGBANK database
	Indication	Refers to the use of drug for the treatment of a particular disease
	IUPAC name	Is the chemical nomenclature of the drug according to IUPAC standards
	State	Indicates the availability of the drug as a treatment.
Study	Date of access	Date of access to the paper source that describes the variant.
	Publication type	Refers to the repository that indexes the paper source (e.g. PubMed)
	Publication year	Date of publication for the source paper
	Type	Type of the study could be: Association, GWAS, Genetic variability study, functional analysis or linkage.
	Title	Title of the source paper

Supplementary Materials File **S3**

African Genomic Medicine Portal Tutorial (Next page)

African Genomic Medicine Portal

Tutorial

Introduction

The African Genomic Medicine Portal (AGMP) functions as a curated resource for researchers around the world who are conducting genomics research on African and African- ancestry populations. The portal may also be useful for individuals working in the health sector, such as healthcare workers, pharmacists, and policymakers, though it was designed as a research tool and should not be used for clinical decisions. The portal functions as a gateway to data relevant for African genomic medicine research, including pharmacogenomics and clinical/disease research, accessing and providing African-specific data from existing resources in an easily accessible manner.

AGMP retrieves and curates data from various resources. The current release contains data retrieved and curated from PharmGKB and DisGeNET.

This tutorial provides a step by step guide to searching data in the Portal.

Search:

1. Access AGMP using the following link: https://agmp.h3abionet.org/. On this page, **four main data categories** will be displayed: **Disease**, **Drug**, **Variant**, and **Gene**, as illustrated in **Figure 1**.

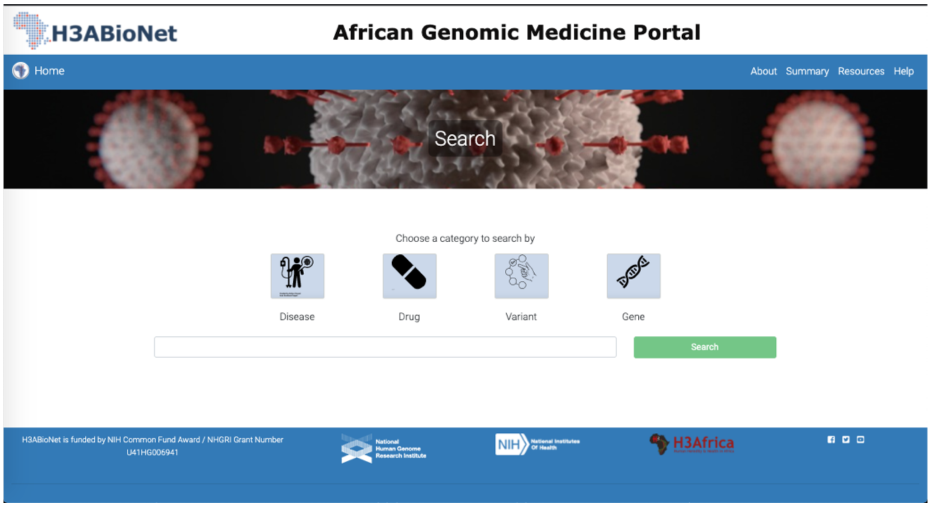

Figure 1. AGMP search page.

2. A user may search based on their preferred data category by selecting the *corresponding data category logo* (Disease, Drug, Variant, or Gene), as illustrated in **Figure 2A-D**. **Results are customized according to the data category** selected.

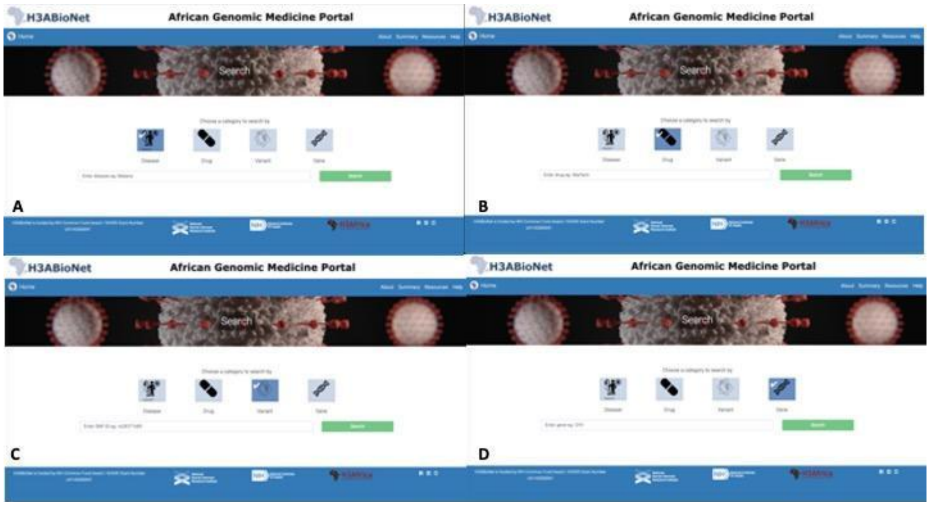

Figure 2. (A) Disease category selected; (B) Drug category selected; (C) Variant category selected; (D) Gene category selected.

3. Enter a search term into the search box and select *Search*. Matching results will appear below the text box, as illustrated in **Figure 3A-D**.

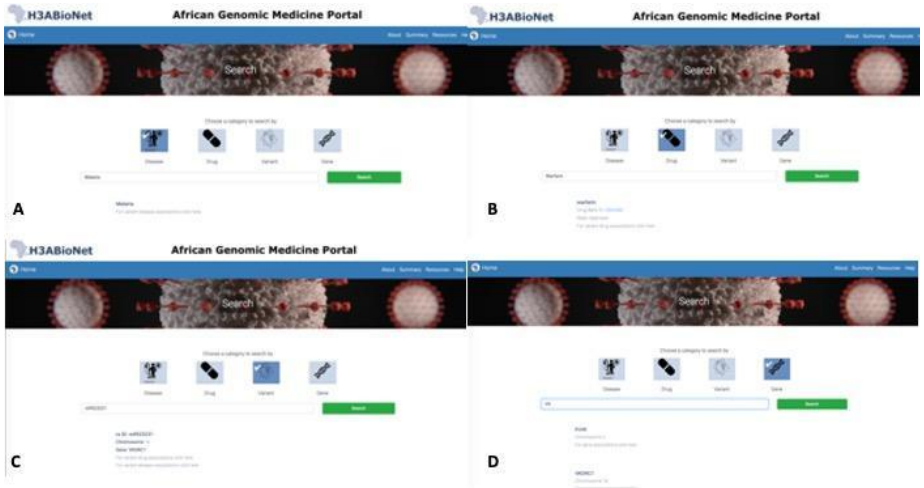

Figure 3. (A) Disease result; (B) Drug result; (C) Variant result; (D) Gene result.

Results:

*NB: Results are discussed by the data search category selected during **Search**.*

1. Disease Results:

1.1. When searching by a disease, a list of relevant results appears below the search box. To proceed to complete results, select the “For variant-disease associations click here” button, as illustrated in **Figure 3A**.

1.2. As illustrated in **Figure 4**, the results page will contain a list of variant-disease associations. Table contents are described in **Table 1**.

Variant Disease associations

Copy CSV Excel Print Search:

Variant	Disease	Gene	Significance	Country	Studies
rs10192428	Malaria	SPATA3	0.00000051	The Gambia	Genome-wide and fine-resolution association analysis of malaria in West Africa.
rs1046089	Malaria	PRRC2A	< 0.0001	The Gambia	A genetic association study in the Gambia using tagging polymorphisms in the major histocompatibility complex class III region implicates a HLA-B associated transcript 2 polymorphism in severe malaria susceptibility.
rs10900585	Malaria	ATP2B4	0.0000000061	Ghana	Genome-wide association study indicates two novel resistance loci for severe malaria.
rs10900585	Malaria	ATP2B4	0.0052	Ghana	Genome-wide association study indicates two novel resistance loci for severe malaria.
rs11335470	Malaria	LINC00944	0.0000904	Tanzania	Novel genetic polymorphisms associated with severe malaria and under selective pressure in North-eastern Tanzania.
rs114169033	Malaria	FRG1-DT	0.000000562	Tanzania	Novel genetic polymorphisms associated with severe malaria and under selective pressure in North-eastern Tanzania.
rs12405994	Malaria	AC092813.1	0.00000082	The Gambia	Genome-wide and fine-resolution association analysis of malaria in West Africa.
rs12788102	Malaria	MMP26	< 0.001	The Gambia, Kenya, Malawi	Imputation-based meta-analysis of severe malaria in three African populations.
rs12788102	Malaria	OR51F1	< 0.001	The Gambia, Kenya, Malawi	Imputation-based meta-analysis of severe malaria in three African populations.
rs12789492	Malaria	MMP26	< 0.001	The Gambia, Kenya, Malawi	Imputation-based meta-analysis of severe malaria in three African populations.

Figure 4. Tabulated Disease result.

Table 1. Descriptions of Disease results column headers.

**Variant**	The genetic variants which have been associated with the disease of interest.
**Disease**	The disease of interest entered in the search box.
**Gene**	The gene in which the variant is located.
**Significance**	The p-value observed for the association in the given study.
**Country**	The country of origin of the research participants.
**Studies**	The study associated with the result.

2. Drug Results:

2.1. When searching by a drug, a list of relevant results appears below the search box. To proceed to complete results, select the “For variant-drug associations click here” button, as illustrated in **Figure 3B**.

2.2. As illustrated in **Figure 5**, the results page will contain a list of variant-drug associations. Table contents are described in **Table 2**.

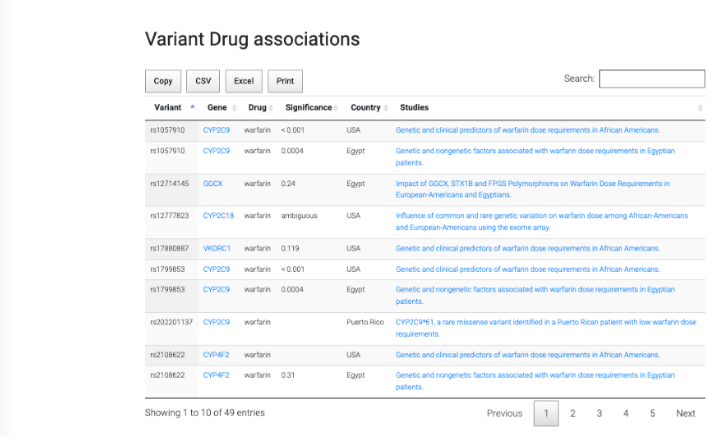

Variant Drug associations

Copy CSV Excel Print Search:

Variant	Gene	Drug	Significance	Country	Studies
rs1057910	CYP2C9	warfarin	< 0.001	USA	Genetic and clinical predictors of warfarin dose requirements in African Americans.
rs1057910	CYP2C9	warfarin	0.0004	Egypt	Genetic and nongenetic factors associated with warfarin dose requirements in Egyptian patients.
rs12714145	GGCX	warfarin	0.24	Egypt	Impact of GGCX, STX1B and FPGS Polymorphisms on Warfarin Dose Requirements in European-Americans and Egyptians.
rs12777823	CYP2C18	warfarin	ambiguous	USA	Influence of common and rare genetic variation on warfarin dose among African-Americans and European-Americans using the exome array.
rs17880887	VKORC1	warfarin	0.119	USA	Genetic and clinical predictors of warfarin dose requirements in African Americans.
rs1799853	CYP2C9	warfarin	< 0.001	USA	Genetic and clinical predictors of warfarin dose requirements in African Americans.
rs1799853	CYP2C9	warfarin	0.0004	Egypt	Genetic and nongenetic factors associated with warfarin dose requirements in Egyptian patients.
rs202201137	CYP2C9	warfarin		Puerto Rico	CYP2C9*61, a rare missense variant identified in a Puerto Rican patient with low warfarin dose requirements.
rs2108622	CYP4F2	warfarin		USA	Genetic and clinical predictors of warfarin dose requirements in African Americans.
rs2108622	CYP4F2	warfarin	0.31	Egypt	Genetic and nongenetic factors associated with warfarin dose requirements in Egyptian patients.

Showing 1 to 10 of 49 entries Previous 1 2 3 4 5 Next

Figure 5. Tabulated Drug result.

Table 2. Descriptions of Drug results column headers.

**Variant**	The genetic variants which have been associated with the drug of interest.
**Drug**	The drug of interest entered in the search box.
**Gene**	The gene in which the variant is located.
**Significance**	The p-value observed for the association in the given study.
**Country**	The country of origin of the research participants.
**Studies**	The study associated with the result.

3. Variant Results:

3.1. When searching by a variant, a list of relevant results appears below the search box. To proceed to complete results, select either the “For variant-

disease associations click here” or the “For variant-drug associations click here” button, as illustrated in **Figure 3C**.

3.2. Based on the selected button, the results page will contain a list of either variant-disease or variant-drug associations, as illustrated in **Figures 4 and 5**. Table contents are described in **Table 3**.

Table 3. Descriptions of Variant results column headers.

**rsID**	The genetic variant of interest entered in the search box.
**Disease**	The diseases associated with the variant of interest.
**Drug**	The drugs associated with the variant of interest.
**Gene**	The gene in which the variant of interest is located.
**Significance**	The p-value observed for the association in the given study.
**Country**	The country of origin of the research participants.
**Studies**	The study associated with the result.

4. Gene Results:

4.1. When searching by a gene, a list of relevant results appears below the search box. To proceed to complete results, select the “For gene associations click here”, as illustrated in **Figure 3D**.

4.2. As illustrated in **Figure 6A-B**, the results page will contain a description section (**6A**), a Pharmacogenomics Associations section (**6A**) and a Disease Associations section (**6B**). Table contents are described in **Table 4.**

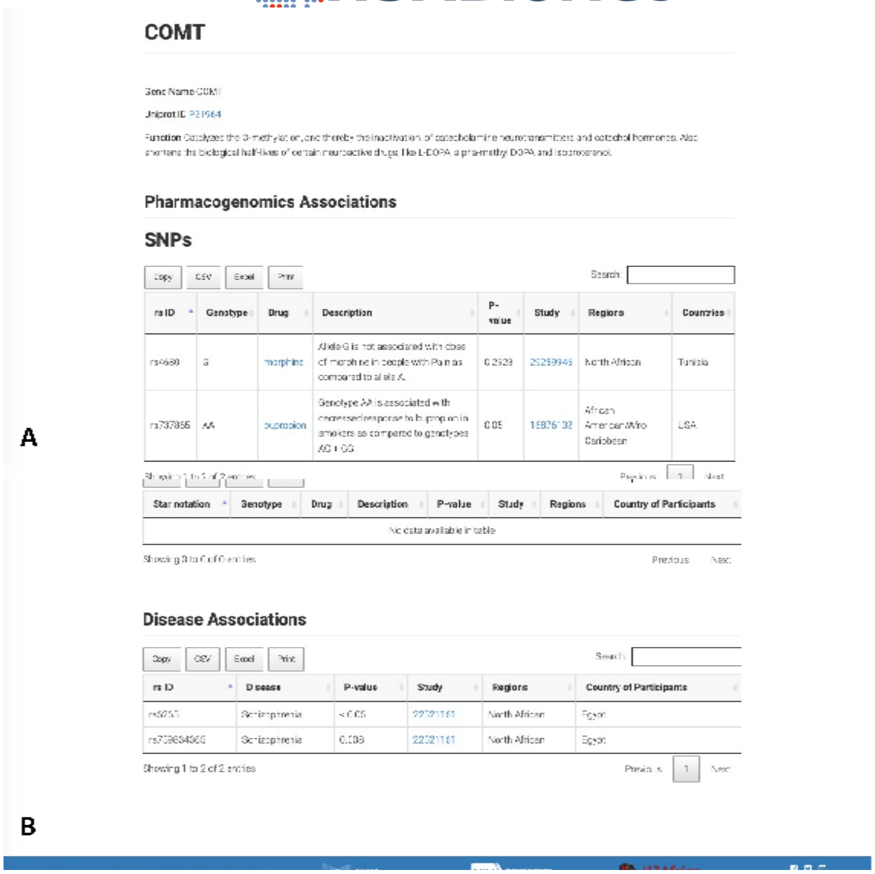

Figure 6. Gene result page.

Table 4. Descriptions of Gene results column headers.

**Variant**	The genetic variants located in the gene which have been studied in African populations
**Genotype**	The genotype of a genetic variant associated with a given drug association.
**Drug**	The drug associated with genetic variant.
**Description**	A description of the drug association.
**P-value**	The p-value observed for a given association.
**Study**	The PMID associated with the associated study.
**Regions**	The region(s) from which the research participants originate.
**Country of Participants**	The Country(ies) from which the research participants originate.
**Disease**	The disease associated with the genetic variant.

4.3. Using the task bars found in each table, the user can access different information on either the disease, drug, variant or gene. Table 1 provides an overview of the different types of information found.

Other Resources

1. When clicking on the **Summary** tab, a summary of the Total Number of Genes, Drugs, Diseases and Variants, included in the portal, is provided. The locations from where the data is derived are also illustrated in a user-friendly map.

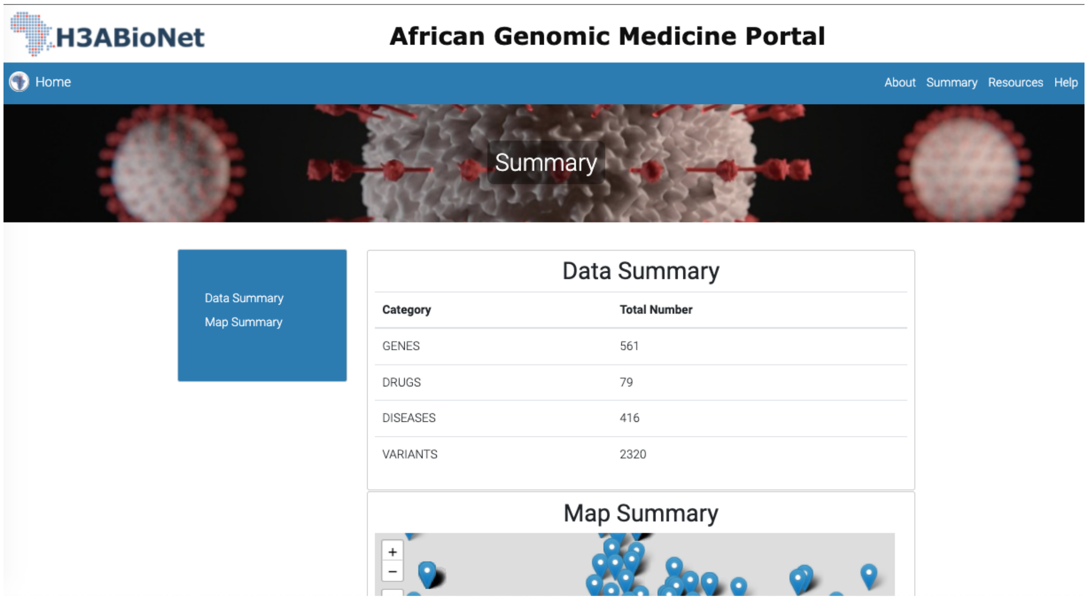

Figure 4: Summary tab.

2. When clicking on the Resources tab, a list of additional H3ABioNet and relevant

   external resources are provided, these include: Databases; Tools & Pipelines; and Online Courses.

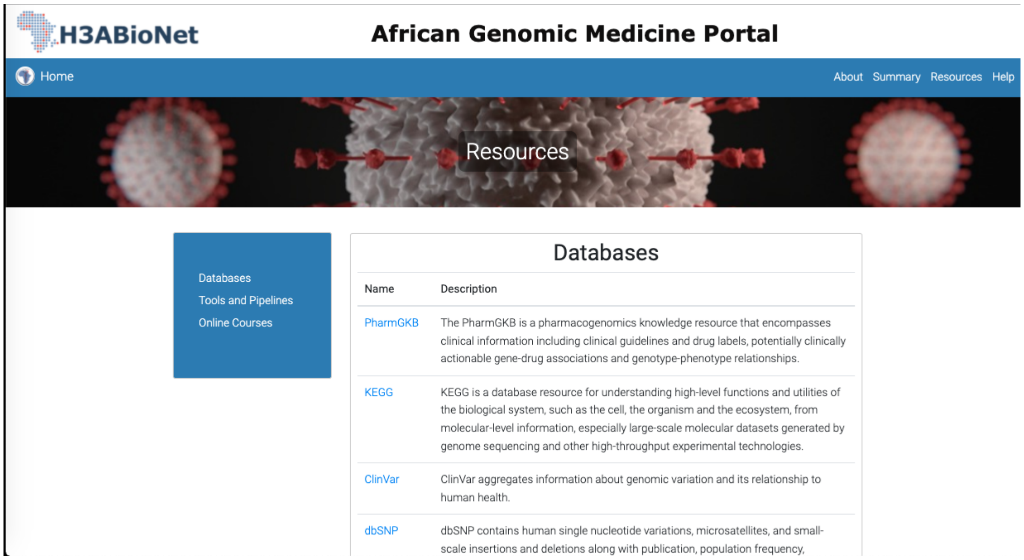

Figure 5: Resources tab.
